# Phonon arithmetic in a trapped ion system

**DOI:** 10.1038/ncomms11410

**Published:** 2016-04-21

**Authors:** Mark Um, Junhua Zhang, Dingshun Lv, Yao Lu, Shuoming An, Jing-Ning Zhang, Hyunchul Nha, M. S. Kim, Kihwan Kim

**Affiliations:** 1 Center for Quantum Information, Institute for Interdisciplinary Information Sciences, Tsinghua University, Beijing 100084, P. R. China; 2 Korea Institute for Advanced Study, Seoul 130-722, Korea; 3 Texas A&M University at Qatar, Education City, P.O. Box 23874, Doha, Qatar; 4 Blackett Laboratory, QOLS, Physics Department, Imperial College London, Exhibition Road, London SW7 2AZ, UK

## Abstract

Single-quantum level operations are important tools to manipulate a quantum state. Annihilation or creation of single particles translates a quantum state to another by adding or subtracting a particle, depending on how many are already in the given state. The operations are probabilistic and the success rate has yet been low in their experimental realization. Here we experimentally demonstrate (near) deterministic addition and subtraction of a bosonic particle, in particular a phonon of ionic motion in a harmonic potential. We realize the operations by coupling phonons to an auxiliary two-level system and applying transitionless adiabatic passage. We show handy repetition of the operations on various initial states and demonstrate by the reconstruction of the density matrices that the operations preserve coherences. We observe the transformation of a classical state to a highly non-classical one and a Gaussian state to a non-Gaussian one by applying a sequence of operations deterministically.

## Abstract (web summary)

Single-quantum level operations are important tools for engineering desired quantum states, but they are inherently probabilistic operations with low experimental success rate. Here, the authors demonstrate near deterministic addition and subtraction of a single phonon of motion in a trapped Yb ion.

## Graphical abstract

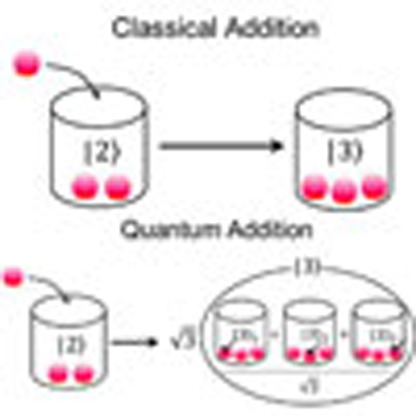

In quantum mechanics, bosonic creation 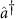 and annihilation 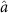 operators bear the following operator relations


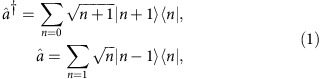


where 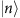 stands for a Fock state of *n* bosons. The proportionality factors 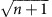 and 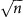 appear due to the symmetric indistinguishable nature of bosons^1^,^2^. Thus, the addition or subtraction in quantum domain involves the modification of the probability amplitude of state due to the excitation *n*-dependent factor. The bosonic annihilation and creation operation have been proposed to be a building block to generate an arbitrary quantum state^3^,^4^, to distill entanglement or non-locality^5^ and to transform a classical state to a non-classical state^6^. In recent times, there have been seminal works to realize the bosonic operations at the single-boson level for the test of foundations and applications of quantum mechanics^7^,^8^,^9^,^10^,^11^,^12^,^13^. However, such bosonic operations are not trace preserving and inherently probabilistic. The probability of success has so far been extremely low in their implementation for photonic fields. A higher probability may be obtained at the expense of lowering the performance fidelity^14^. Owing to this, a repetition of these operations has been limited.

The conventional addition and subtraction of a particle can be written as


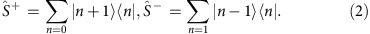


The addition 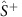 takes the *n* particle state to the (*n*+1) state, whereas the subtraction operation 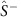 brings the *n* state to the (*n*−1) state without incurring additional factors. Seeing the form of the operations in equation (2), we immediately recognize that 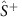 is a deterministic process while 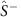 may not be, as it is not possible to subtract a particle from vacuum |0〉. When the vacuum component of the initial state is small, the subtraction can be done near-deterministically. In recent times, there have been theoretical proposals of the operations (equation (2)) for the generation of an arbitrary quantum state^15^, the measurement of vacuum^16^, the transformation to a non-classical state^17^ and the amplification of a quantum state^18^. In particular, such arithmetic operations form an important component of a qubit gate operation for ions in a harmonic potential^19^. The operations (equation (2)) were also suggested as the elements of a phase operator^20^. Beyond the quantum-state engineering, the subtraction can be used for the sub-Doppler cooling in the trapped ion system^21^.

In this study, we experimentally demonstrate deterministic addition and near-deterministic subtraction of a bosonic particle, in particular a phonon of a ^171^Yb^+^ ion trapped in a harmonic potential. We realize the operations by coupling phonons to an auxiliary two-level system, so called the hybrid scheme of discrete and continuous variable^22^, and applying a transitionless quantum-driving scheme. We perform the operations on superpositions of Fock states and coherent states, and we demonstrate that our single-phonon operations are (near) deterministic and preserve coherence. By applying a sequence of operations deterministically, we show that classical states are turned into non-classical ones manifesting highly sub-Poissonian statistics and negativity in the Wigner function.

## Results

### Experimental implementations

**Figure 1:**
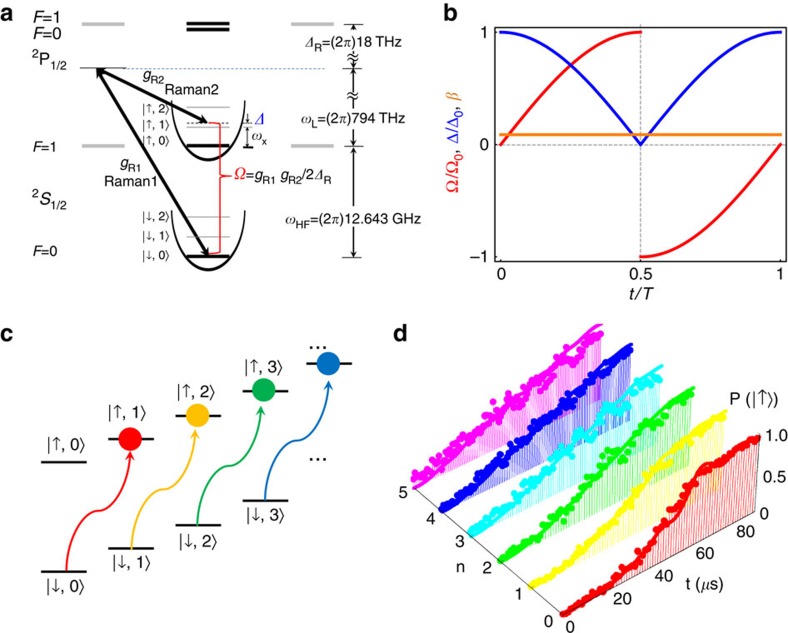
Experimental scheme and dynamic and adiabatic transition by anti-Jaynes–Cummings interaction. (**a**) ^171^Yb^+^ system in a harmonic potential. The qubit level in *S*_1/2_ manifold, 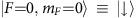 and 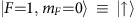 are coupled by the Raman laser beams, where the beat-note frequency is near resonant to the qubit levels, *ω*_HF_. When the beat-note frequency is tuned to ∼*ω*_HF_+*ω*_X_, the scheme produces the anti-Jaynes–Cummings interaction or blue-sideband transition. We denote Ω as the Rabi frequency on the qubit transition and Δ is the frequency difference between the beat-note frequency of Raman beams and *ω*_HF_+*ω*_X_. The Raman beams are realized by picosecond pulse train similar to the scheme in ref. 45. (**b**) The controls of experimental parameters of Ω and Δ for the uniform blue-sideband transition. Naturally, the Rabi frequency of the blue-sideband transition is dependent on the motional quantum number *n*. To remove the *n*-dependency, we control Ω and Δ as the red and blue curves such as Ω(*t*)=Ω_0_[sign(*T*/2−*t*)sin(*πt*/*T*)+*iβ*] and Δ(*t*)=Δ_0_sign(*T*/2−*t*)cos(*πt*/*T*). The phase *iβ* in Ω is the counter-diabatic term to suppress the transition during the evolution. Here Ω_0_=(2*π*)38.5 kHz, *β*=0.075 and Δ_0_=1.6Ω_0_. (**c**) The basic operation of the uniform blue-sideband transition without the 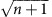 dependence by the pulse shaping of **b**. (**d**) The experimental demonstration of the uniform blue-sideband transitions. The total time to execute the transitions is 91 μs for any 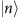 (*n*=0, 1, ... 5).

We implement the 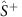 and 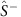 operations of equation (2) on a vibrational mode of frequency *ω*_X_ for a single trapped ^171^Yb^+^ ion in a three-dimensional harmonic potential^23^ through its interaction with the two-level system of atomic energy levels. The harmonic potential is generated by an oscillating electric field in the radial axis with trap frequency *ω*_X_=(2*π*)2.8 MHz. The two-level system is represented by two hyperfine states 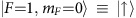 and 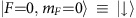 of the *S*_1/2_ manifold with the transition frequency *ω*_HF_=(2*π*)12.6428 GHz. As shown in Fig. 1a, the anti-Jaynes–Cummings (aJC) interaction or blue-sideband transition, 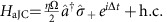, is realized by the stimulated Raman laser beams with beat-note frequency (*ω*_R1_−*ω*_R2_)=(*ω*_HF_+*ω*_X_)+Δ. Here, Ω is the Rabi frequency of the two-level system, 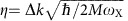 the Lamb–Dicke parameter, Δ*k* the net wave vector of the Raman laser beams and *M* the mass of ^171^Yb^+^ ion. The aJC coupling produces the transition between 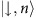 and 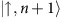 with the oscillation frequency of 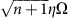, where the 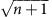 factor comes from the fundamental property of 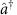 and 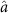 operators in equation (1). Therefore, the application of the simple aJC interaction does not transfer 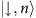 to 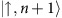 in an *n*-independent manner at a fixed duration of time.

The full population transfer independent of the initial motion state, the uniform blue-sideband transition, 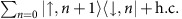, can be obtained by the application of the stimulated Raman adiabatic passage^24^,^25^,^26^. The adiabatic scheme provides a robust transfer against the variations in the transition rate either from the intensity change of the control Raman beams or from the property of the transitions^25^. Therefore, a properly designed adiabatic passage would allow a decent state transfer for a wide range of phonon number states through the aJC interaction, despite the 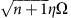 dependence. In the adiabatic passage, typically *η*Ω slowly increases at the beginning and decreases at the end, that is, Ω(*t*)=Ω_0_ sin(*πt*/*T*), whereas the detuning Δ changes according to Δ(*t*)=Δ_0_ cos(*πt*/*T*), where *T* is the total transfer time, across the resonance. However, for the applicability of the scheme to a wide range of initial phonon numbers with high fidelity, we should set Δ_0_ as high as 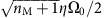, where *n*_M_ is the largest phonon number for the transfer and should fulfill the adiabatic condition, 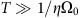. In our experimental conditions, the reasonable duration *T* for such an adiabatic transfer is around 21 times that of *π*-pulse duration for the blue-sideband transition of the ground state *π*/*η*Ω_0_, when we include up to the maximum phonon number *n*_M_=6.

In recent times, the transitionless quantum-driving scheme^27^,^28^,^29^,^30^ has been developed to speed up the adiabatic control. When the transfer is non-adiabatic, it introduces an additional term in the Hamiltonian of the instantaneous basis, which is 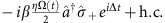, where *β* is in the order of *π*/*η*Ω_0_/2*T*, the ratio between the *π*-pulse duration and the total operation time. By adding a counter-diabatic term in the control, we can suppress the non-adiabatic excitation with a reasonable speed up over the adiabatic passage. For the aJC interaction, the optimal values of Δ_0_ and *β* are dependent on the phonon number *n* for the given Ω_0_. In our experiment, we optimize Δ_0_ and *β* for the case of the geometric average of the minimum and maximum phonon number, 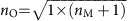. By doing this, we are able to reduce the total duration of the operation from 21 to 7 times *π*/*η*Ω_0_ without sacrificing the fidelity of the rapid adiabatic passage for the same range of phonons *n*_M_=6.

To rigorously achieve the uniform blue-sideband operation 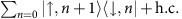, it is important not only to increase the phonon number but also to preserve the relative phases between component states of the quantum state. For our previous realization^23^, the different extra phases were accumulated depending on the phonon number of the initial state, which prevented from keeping the initial phase coherences (see ‘AC Stark shift compensation' in Methods). In this study, we have developed a sequence of operations compensating the phonon-number-dependent phases based on the spin-echo principle. As shown in Fig. 1b, we invert the sign of Ω and reverse the control of Δ in the middle of the sequence of the operation, which symmetrizes the whole operation and produces the accumulated phases of opposite signs before and after the inversion and reverse. Therefore, the total phases are cancelled out at the end of the operation (see ‘AC Stark shift compensation' in Methods).

### Conventional phase-coherent addition

**Figure 2:**
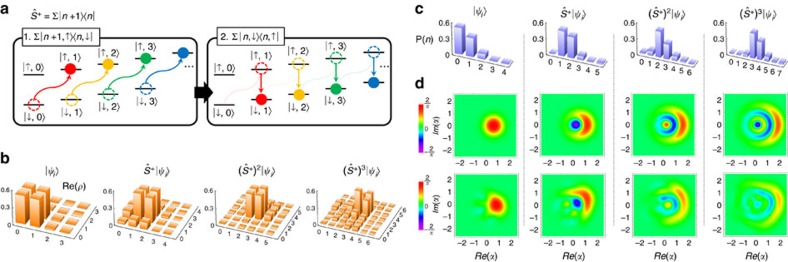
Schematic diagram and experimental results for the phonon addition. (**a**) Implementation of addition is composed of a *π*-pulse of uniform blue-sideband transition 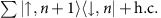, followed by a *π*-pulse of carrier transition 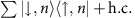 This realizes the addition operation 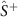 of equation (2). (**b**) Additions on a superposition state 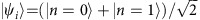 clearly shows the capability of keeping coherence. The reconstructed density matrices, only the real part of them, indicate the fidelity 0.99 (<0.01) of the initially prepared state and those of the final states 0.96(0.01), 0.92(0.01) and 0.87(0.01) after 1, 2 and 3 times addition, respectively. The purities of the output states are 0.92(0.01), 0.81(0.03) and 0.71(0.06), respectively. The numbers in the parentheses represent the sizes of error estimated by the maximal-likelihood methods (see Methods). (**c**) Phonon distributions after the addition on a coherent state 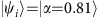. Our addition operations shift up the populations on phonon numbers while keeping the variance the same, making the variance over average phonon number 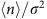, which is initially set to 1, reduce to 0.43, 0.39 and 0.26 as the average phonon number is increased by 1, 2 and 3, respectively. (**d**) Wigner functions of the coherent state 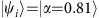 after performing the addition operation *n* times (*n*=0, 1, 2 and 3). The upper figures are theoretical and the lower figures are experimental. Observed negative values in the Wigner function proves the production of non-Gaussian state. The fidelities are reduced from 0.97(0.01) for the initial state to 0.87(0.01) (one single-phonon addition), 0.84(0.01) (two additions) and 0.85(0.02) (three additions), and purities are changed from 0.99 to 0.93(0.02), 0.93(0.02) and 0.80(0.03), respectively.

We implement the coherent addition operation 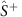 in equation (2) by applying the uniform blue-sideband transfer followed by the *π*-pulse of carrier transition as shown in Fig. 2a. By this operation, we add single phonons deterministically, independent of the initial number of phonons. To manifest the coherence property of the addition operations, we prepare an initial state 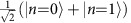, apply the operations up to three times and measure the density matrices of the resulting phonon states. As shown in Fig. 2b, the coherences represented by the off-diagonal terms of the density matrix clearly remain after the multiple addition processes up to three times.

We also consider an initial coherent state of the amplitude *α*=0.81, which is achieved by displacing the vacuum. The density matrix is reconstructed (see Fig. 2c,d) by using the iterative maximum-likelihood algorithm^31^ on the phonon number distributions for eight different angles (see ‘Reconstruction of the phonon density matrix' in Methods). The phonon number distributions are obtained by observing the time evolutions of the standard blue-sideband transitions, similar to the direct reconstruction scheme of the phonon density matrix^32^. One immediate consequence of adding single phonons on the coherent state is the production of sub-Poissonian phonon statistics, because the addition increases the average phonon number but not the shape of the distribution nor the variance. We observe that the ratios between the variance and the average phonon number reduce from 1 to 0.43, 0.39 and 0.2, after 1–3 single-phonon additions, respectively. Applying the first addition operation, we detect negativity in the Wigner function as shown in the second column of Fig. 2d. It is important to note that the addition operation, which converts a coherent state to a highly non-Gaussian and non-classical state, is deterministic unlike the case of 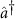 operation^9^,^10^,^11^. There is a technical limit in the number of additions we can apply, owing to the validity of the adiabatic approximation and the heating process of phonons^33^. Under this limitation, we could perform the operations three times without the significant loss of fidelity. As shown in Fig. 2d, the experimental results and the theoretical predictions for the Wigner functions are in agreement, except the shift of the orientation, which would come from the phase offset between the preparation and the analysing pulses. This is significant in comparison with the photonic realization of bosonic operations of single-photon creation and annihilation^11^. We detect negativity in the Wigner function as shown in the second column of Fig. 2, where we obtain the Wigner function of the state from the reconstructed density matrix.

### Conventional subtraction operation

**Figure 3:**
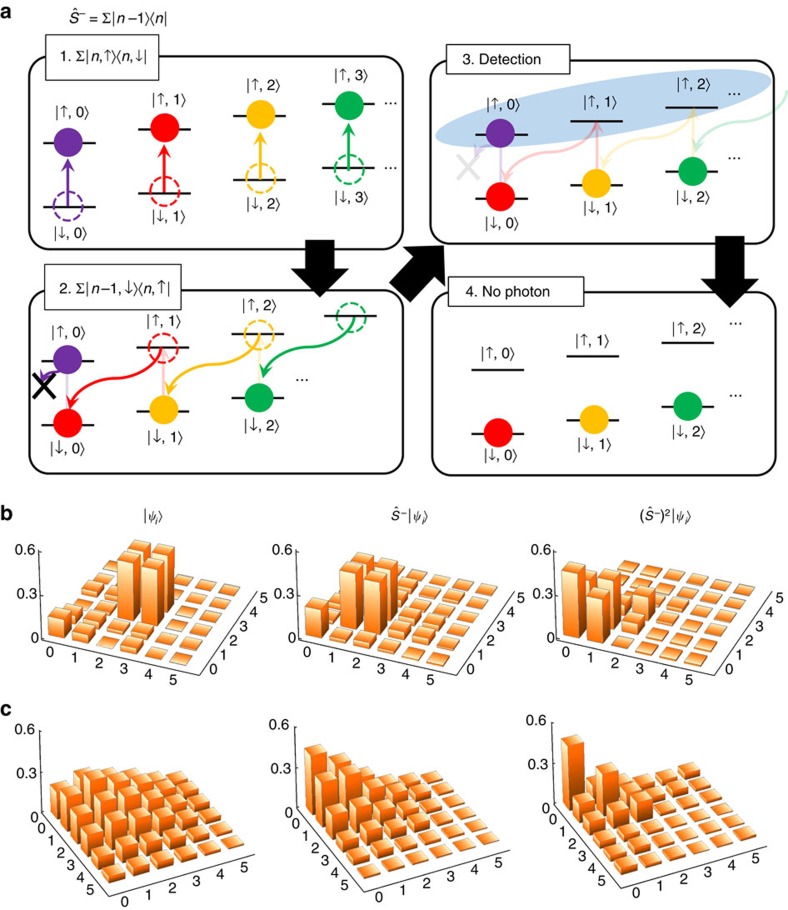
Schematic diagram and experimental results of phonon subtraction. (**a**) Sequence of subtraction operations: the sequence of the operations for addition is reversed, that is, a *π*-pulse of carrier transition followed by a *π*-pulse of adiabatic blue-sideband transition. This takes the phonon state from 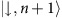 to 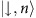, except 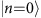 where 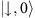 transfers to 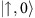. The 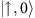 state is abandoned by the conditional measurement after the fluorescence detection, which only collects non-zero phonon state data without fluorescence. (**b**) Subtraction on a superposition state 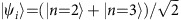. The population is reduced and the coherence is conserved. The initial fidelity and purity of the state are 0.83(0.02) and 0.73(0.03). The fidelities are changed to 0.77(0.02) and 0.83(0.01) after 1 and 2 times subtraction, respectively. The purities become 0.65(0.02) and 0.75(0.02). In the preparation and displacement operations for the superposition states with fluorescent detection, the zero components are increased due to unexpected experimental imperfections, which accidentally increase the fidelity and purity for the state 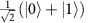. (**c**) Subtraction from an initial coherent state *α*=1.2. The initial fidelity of the state is 0.96(0.01) and the fidelities are reduced to 0.92(0.01) and 0.66(0.01) after 1 and 2 times subtraction, respectively.

The subtraction operation 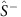 in equation (2) is realized by reversing the sequence of the addition operation, that is, the application of the *π*-pulse of carrier transition and the uniform blue-sideband transfer, followed by the fluorescence detection as shown in Fig. 3a. The zero phonon state 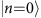 is eliminated after the subtraction, which is implemented by the conditional measurement in our experimental scheme. After the detection sequence, we only collect the data with no fluorescence, which has the success rate given by the probability of the non-zero phonon states. We examine the performance of the subtraction operation with an initial phonon superposition state 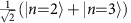. As shown in Fig. 3b, the subtraction operation reduces the phonon excitation by one quanta. After the second application of the subtraction, the off-diagonal terms of the density matrix are significantly reduced, which shows the current limit in experiments due to heating of the system. We also prepare a coherent state 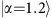 and apply the subtraction twice as shown in Fig. 3c, which shows qualitatively that the subtraction operation works for any initial quantum state. The subtraction operation can squeeze a coherent state, which is different from annihilation 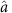 that has the coherence state as its eigenstate. However, our experimental precision is not high enough to observe the squeezing effect.

### Commutation relation of addition and subtraction operations

**Figure 4:**
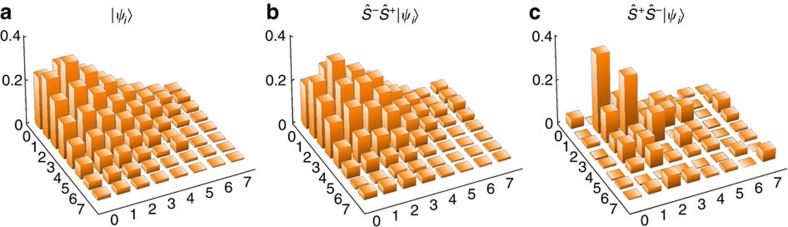
Experimental results after addition-then-subtraction and subtraction-then-addition. Only the real part of experimentally measured density matrices is shown (**a**) for an initial coherent state 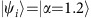, (**b**) single-phonon added-then-subtracted 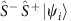 state and (**c**) single-phonon subtracted-then-added 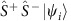 state. (**b**) The state after addition-then-subtraction is basically identical to the original state. The fidelity of the 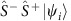 state to the original state 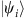 is 0.97(0.01) and the purity is 0.96(0.01). (**c**) The state after subtraction-then-addition is not the same as the original state, because the vacuum component is thrown away during the projective measurement. The small population in zero component mainly comes from the imperfection of the fluorescence detection and heating of the system, which is in good agreement with numerical simulation.

We study experimentally how the quantum states are changed depending on the order of the addition and subtraction for an initial coherent state 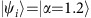. If we add and then subtract 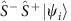, the state after the sequence is the same as the original one, as there is no amplitude modification. For the case of subtraction-then-addition 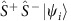 the final state does not have vacuum component, because a vacuum component is annihilated by 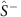 and cannot be restored by 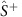. Figure 4b shows the experimental result of 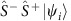, which is basically identical to the initial state of Fig. 4a. Figure 4c shows the result after the operation of 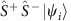, where there is no significant vacuum component in the density matrix. The vacuum component is not perfectly absent because of the detection error during the projective measurement based on the atomic fluorescence and heating of the system. The fluorescence detection duration is comparable to the motional coherence time of our system, which makes the off-diagonal part of the final state suppressed significantly (see ‘Error analysis' in Methods). Our experimental result is well in line with the non-commuting relation of the Susskind–Glogower's phase operators, that is, 
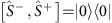
 (ref. 20).

## Discussion

Owing to the capability of deterministically generating a non-classical and non-Gaussian states, the conventional addition and subtraction operations provide an efficient scheme for quantum state engineering^34^,^35^. The current work can also be a stepping stone to realize the ion qubit gate operation as proposed by Schneider *et al*.^19^ without having to worry about long operation times and loss of coherences. This scheme may be further applied to various quantum optics setups such as cavity quantum electrodynamics and optomechanics. We note a theoretical scheme that the non-Gaussian information of a phonon state would be transferred to that of photon through phonon–photon coupling^36^,^37^. It has also been discussed that the conventional arithmetic addition and subtraction can be used to measure the vacuum state without disturbing the state^16^ and therefore it can be directly applied to construct the *Q*-function of a quantum state of motion. We have noted that an arithmetic subtraction was performed for a photonic system^38^.

## Methods

### AC Stark shift compensation

The AC Stark shift in the adiabatic operations mainly comes from the off-resonant coupling to the carrier transition, the transition between *S*_1/2_↔*P*_1/2_ states of ^171^Yb^+^ ion and the other radial motional mode (*ω*_Y_≈*ω*_X_+(2*π*)0.4 MHz). The dominant AC stark shift comes from the carrier transition of frequency 
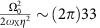
 kHz with Ω_0_=(2*π*)38.5 kHz and the Lamb–Dicke parameter *η*=0.089. The amount of the shift brought by the Y mode is given by 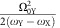, that is, ∼20 times smaller than that from the carrier coupling. The AC stark shift between qubit states from the Raman laser beams due to *S*_1/2_↔*P*_1/2_ transition is 
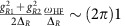
 kHz, where *g*_R1_ and *g*_R2_ are the coupling strengths of Raman 1 and Raman 2 beams, respectively, and the Δ_R_=(2*π*) 18 THz is the detuning from the level of ^2^*P*_1/2_.

We consider total AC stark shift as the form of 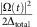, where Δ_total_ is the detuning effectively including all the possible origin of AC stark shifts discussed above. We obtain 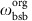 and Δ_total_ by fitting the several points of 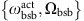 with the equation 
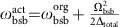
. The actual frequency of blue-sideband 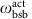 is measured by observing the resonant excitation. Including the AC stark shift, the actual waveform of Ω(*t*) that we apply on our arbitrary waveform generator is as follows:


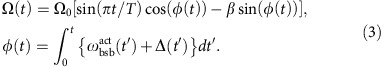


Here, Δ(*t*′)=Δ_0_ cos(*πt*/*T*) and we note that the imaginary part in original form of Ω(*t*)=Ω_0_[sin(*πt*/*T*)+*iβ*] is changed to sin-wave, which has the 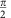 phase difference. Here, *φ*(*t*) is calculated as follows:


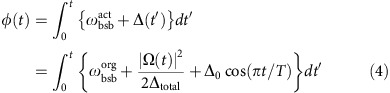



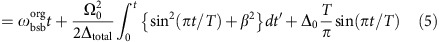



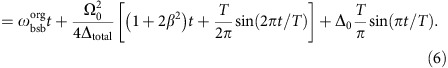


### Reconstruction of the phonon density matrix

We use an iterative algorithm in ref. 31 for the reconstruction of a quantum state. It consists of a maximum-likelihood estimation solved by the expectation-maximization algorithm followed by a unitary transformation of the eigenbasis of the density matrix *ρ*.

Based on the measured phonon distribution *f*_*n*_ by *N* measurements after a displacement of the quantum state, we aim to get real probabilities 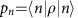 that are as close to the observed frequencies *f*_*n*_ as possible, subject to the maximum-likelihood functional


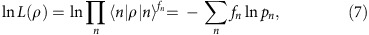


from which we reconstruct *ρ*. This likelihood functional can be interpreted as a linear and positive problem in the classical signal processing:


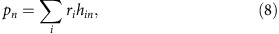


where *r*_*i*_ is the eigenvalues of *ρ* and *h*_*in*_ is a positive kernel. We can solve this linear and positive problem with the expectation-maximization algorithm^39^,^40^:


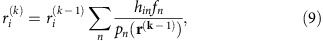


which is initially set to a positive vector **r** 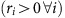. This is repeated for different displacements.

The second part of the reconstruction scheme aims at getting the eigenbasis diagonalizing the density matrix. This part consists of two steps: reconstruction of the eigenvectors of *ρ* in a fixed basis and rotation of the basis using a unitary transformation


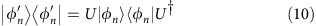


with the infinitesimal form 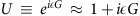 and 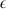 is a small positive real number. *G*=*i*[*ρ*, *R*] is chosen as a Hermitian generator of the unitary transformation, where *R* is a semipositive definite Hermitian operator 
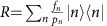
.

Starting from some positive initial density matrix *ρ*, we continue repetition of first finding eigenvalues *r*_*i*_ using the expectation-maximization iterative algorithm (equation (9)) and then finding eigenvectors *φ*_*i*_ by unitarily transforming the old ones. The likelihood of the estimate *p*_*n*_ is increased and we finally reach to determine the density matrix


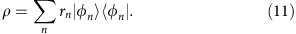


### Error analysis

Dominant error comes from the phonon heating process caused by the electric-field noise from the trapped electrodes^41^. Heating decreases the Fock state preparation fidelity and affects the adiabatic blue sideband process. Its time evolution is known to be described by^41^,^42^:


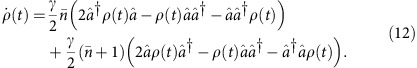


where *γ* is the coupling strength between the ion motion and the thermal reservoir and 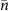 is the average phonon number for the thermal reservoir. In our experimental setup, the heating rate 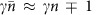 is 150 Hz. It can be reduced by using a large trap, cleaning the electrodes^43^ (equivalent to reducing *γ*) or cooling the trap (equivalent to reducing 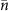) (ref. 44).

Error may be caused by fitting, as some noise in the blue-sideband curves may be recognized as a high phonon population. Although the Fock state preparation error will be involved in the transition probability, fortunately the error for a small *n* is relatively insignificant and the population mainly resides in small *n*-values for the initial states we prepared in the experiments.

There are some other small errors that we did not calculate or simulate, including the fluctuation of the trap frequency ∼0.1 kHz coming from ∼2% intensity fluctuation of Raman laser and ∼1 kHz trap frequency fluctuation coming from sudden changes of the ion position. We see how much our experimental results are modified when comparing with the simulations^23^.

## Additional information

**How to cite this article:** Um, M. *et al*. Phonon arithmetic in a trapped ion system. *Nat. Commun.* 7:11410 doi: 10.1038/ncomms11410 (2016).
